# miRNA‐215‐5p suppresses the aggressiveness of breast cancer cells by targeting Sox9

**DOI:** 10.1002/2211-5463.12733

**Published:** 2019-10-22

**Authors:** Jia Bao Gao, Ming Nan Zhu, Xiao Liang Zhu

**Affiliations:** ^1^ Department of Vascular Breast Surgery People’s Hospital Affiliated to Nanchang University Jiangxi China

**Keywords:** breast cancer, growth, metastasis, miRA‐215‐5p, Sox9

## Abstract

Several studies have shown that miR‐215‐5p acts as a tumor suppressor in certain cancers, but its role in the progression and metastasis of breast carcinoma remains incompletely understood. Herein, we prove that miR‐215‐5p is substantially down‐expressed in breast carcinoma as compared with nontumor tissue. Up‐regulation of miR‐215‐5p inhibits the aggressive abilities of breast carcinoma cells *in vitro*. We performed luciferase reporter tests to show that SRY‐Box 9 (Sox9) is the target of miR‐215‐5p; as predicted, Sox9 depletion replicates the suppressive effects of miR‐215‐5p on breast carcinoma cells, and overexpression of Sox9 rescues the effects of miR‐215‐5p on breast cancer cell progression. In addition, a xenograft model assay was used to reveal that miR‐215‐5p inhibits breast cancer cell growth and metastatic potential *in vivo*. Overall, these results imply that miRNA‐215‐5p suppresses the aggressiveness of breast cancer cells through targeting Sox9.

**Abbreviations**
GAPDH: glyceraldehyde‐3 phosphate dehydrogenase
IHC: immunohistochemistry
miR‐ctr: miRNA negative control
MTT: 3‐(4,5‐dimethyl‐2‐thiazolyl)‐2,5‐diphenyl‐2‐H‐tetrazolium bromide
siSox9: small interfering RNA targeting Sox9
Sox9: SRY‐Box 9
wt: wild‐type

Breast carcinoma remains one of the deadliest malignancies among gynecologic oncology 1. Despite surgical resection combined with radiotherapy or chemotherapy being effective in improving the prognosis of patients with breast carcinoma, those patients who have distant metastasis and recurrence after the surgical resection have high mortality rates 2. Accordingly, the identification of molecules involved in breast cancer tumorigenesis and metastasis is helpful for developing new potential targets to treat breast cancer 3.

miRNAs, belonging to the endogenous noncoding RNA family, regulate the level of target protein via interacting with the 3'‐UTR of the target 4, 5. The dysfunction of miRNAs has a close association with the growth and progression of tumors, and several miRNAs act as tumor suppressors or oncogenes in different kinds of tumors 6, 7, 8. Earlier research has shed light on the fact that miR‐215 is significantly down‐expressed in human breast carcinoma and acts as an anticancer gene 9, 10, 11. However, the exact function of miR‐215‐5p in breast carcinoma cell growth, as well as metastasis, has not been investigated.

In this research, we demonstrate that miR‐215‐5p is substantially down‐expressed in breast carcinoma, and its level has a negative correlation with the development of breast carcinoma. The *in vitro* experiments suggest that upregulation of miR‐215‐5p significantly restrains the proliferation and metastasis of breast cancer MCF‐7 and MDA‐MB‐231 cells. *SRY‐Box 9* (*Sox9*) is certified as the target gene of miR‐215‐5p; furthermore, the inhibition effects of miR‐215‐5p on breast cancer cell migration ability and invasiveness are phenocopied by Sox9 slicing. Altogether, these findings verify that miR‐215‐5p represses the growth, migration and invasion of breast cancer cells by modulating Sox9.

## Materials and methods

### Breast carcinoma cell lines and tissues

The normal mammary epithelial cell line, MCF‐10A, and breast carcinoma cells (MDA‐MB‐468, MDA‐MB‐231 and MCF‐7) were purchased from the Shanghai Institute of Biological Sciences (Shanghai, China). Cells were cultured using RPMI 1640 or Dulbecco’s modified Eagle’s medium (Thermo Fisher Scientific, Waltham, MA, USA) that contained 10% FBS (Thermo Fisher Scientific) and maintained in an incubator with 5% CO_2_ at 37 °C. From August 2010 to August 2017, 39 cases of breast carcinoma tissues and corresponding normal tissues were obtained from the People’s Hospital Affiliated to Nanchang University. Written informed consent was obtained from patients before surgery. This study was approved by the Research Ethics Committee of the People’s Hospital Affiliated to Nanchang University. The study conforms to the Code of Ethics of the World Medical Association (Declaration of Helsinki) printed in the *British Medical Journal* (18 July 1964). The characteristics of patients are summarized in Table S1.

### Cell transfection

miR‐215‐5p mimic (100 nm), small interfering RNA targeting Sox9 (siSox9; 400 nm), miRNA negative control (miR‐ctr) and small interfering RNA control (siCon) were obtained from Qiagen (Hilden, Germany). A Sox9 expression construct was constructed by subcloning the PCR‐amplified full‐length Sox9 cDNA into pMSCV retrovirus plasmid (Guangzhou FulenGen Co., Ltd., Guangzhou, Guangdong, China). miR‐215‐5p or siSox9 was transfected into MDA‐MB‐231 or MCF‐7 cells for 48 h using the Lipofectamine 3000 kit (Thermo Fisher Scientific).

### MTT assay

Cells (5 × 10^3^) were plated into 96‐well plates. A 100‐μL 3‐(4,5‐dimethyl‐2‐thiazolyl)‐2,5‐diphenyl‐2‐H‐tetrazolium bromide (MTT) solution was plated into each well after 1, 2, 3, or 4 days, respectively. The absorbance value was detected at 450 nm.

### Colony formation

Cells (1 × 10^3^) were cultured into a six‐well plate. After 2 weeks, the cell colonies were fixed with 70% EtOH and stained by crystal violet. The number of cell colonies was counted under a microscope (Olympus, Tokyo, Japan) 12.

### Migration assay

Cells (2 × 10^5^) were added to a six‐well plate overnight. Then a straight line of damage was made with a 100‐μL pipette tip. Cells were cultured with FBS free medium for 24 h. Pictures were taken at the times of 0 and 24 h. The percentage of wound healing = (0 h width of wound − 24 h width of wound)/0 h width of wound × 100%.

### Invasion assay

A total of 200 μL of cells (5 × 10^4^) was added to the upper chamber of Tranwells (8‐μm pore size), which was coated with Matrigel (BD, Waltham, MA, USA). A total of 600 μL of medium (20% FBS) was plated to the lower chamber. After 24 h, the Transwell membrane was subsequently fixed in methanol (75%) and stained using crystal violet (1%). The invaded cell was counted under a microscope (Olympus).

### Quantitative real‐time PCR analysis

The preparation of total RNA was carried out using TRIzol (Thermo Fisher Scientific). miRNAs were extracted using the PureLink™ miRNA Isolation Kit (Thermo Fisher Scientific). The synthesis of cDNA was carried out using a PrimeScript RT reagent kit (Takara Bio, Tokyo, Japan). Quantitative real‐time PCR was performed using SYBR Premix Ex Taq II (Takara Bio) and the iQ5 real‐time detection system (Bio‐Rad Laboratories, Hercules, CA, USA). The relative expression of a gene was measured using the $2^{-\Delta \Delta C_{t}}$ method. The relative expression of miR‐215‐5p and Sox9 was normalized to the U6 and glyceraldehyde‐3 phosphate dehydrogenase (GAPDH) level, respectively. The primers in quantitative real‐time PCR were as follows: miR‐215‐5p, 5′‐UAUGGCUUUUUAUUCCUAUGUGA‐3′; Sox9, 5′‐AGTACCCGCATCTGCACAAC‐3′ (forward) and 5′‐ACGAAGGGTCTCTTCTCGCT‐3′ (reverse); GAPDH, 5′‐ATGGGACGATGCTGGTACTGA‐3′ (forward) and 5′‐TGCTGACAACCTTGAGTGAAAT‐3′ (reverse).

### Luciferase reporter gene assay

The plasmid pGL3 that contained the wild‐type (wt) 3′‐UTR of Sox9 or mutant type of pGL3‐Sox9 3′‐UTR combination with miR‐215‐5p or miR‐ctr was cotransfected into MDA‐MB‐231 or MCF‐7 cells using Lipofectamine 3000 (Thermo Fisher Scientific). The luciferase reporter gene assay was carried out using the Luciferase® Reporter Assay System (Promega, Madison, WI, USA).

### Immunoblotting analysis

Total proteins were abstracted using radioimmunoprecipitation assay lysis (Beyotime, Nanjing, Jiangsu, China). The proteins were separated using 10% SDS/PAGE and then transferred onto the poly(vinylidene difluoride) membrane (Millipore, Braunschweig, Germany). The poly(vinylidene difluoride) membrane was incubated with Sox9 (ab185230, 1 : 1000; Abcam, Cambridge, UK) or GAPDH antibodies (sc‐47724, 1 : 1000; Santa Cruz Biotechnology, Dallas, TX, USA) overnight at 4 °C followed by incubating with goat anti‐rabbit IgG conjugated to horseradish peroxidase (BS13278, 1 : 10 000; Bioworld Technology, Inc., Nanjing, Jiangsu, China) for 2 h. Bands were visualized using the electrochemiluminescence system (Millipore).

### Xenograft model

BALB/c nude mice (*n* = 6 in each group) were inoculated with 100 μL of MDA‐MB‐231 cells (1 × 10^6^). The tumor volume in each group was recorded each week and was calculated (tumor volume = 0.5 × width^2^ × length). Finally, mice were sacrificed and tumor tissues were subjected to immunohistochemistry (IHC) staining. This study was approved by the Ethics Committee (No. 20161009) of the People’s Hospital Affiliated to Nanchang University, Nanchang, Jiangxi Province, China.

### Pulmonary metastasis model

MDA‐MB‐231 cells (5 × 10^6^) were injected into BALB/c nude mice (*n* = 6 in each group) via the tail vein. After 8 weeks, nude mice were sacrificed. In addition, the lungs were conducted for the hematoxylin and eosin staining. The animal experiment received approval from the Institutional Animal Care and Use Committee at the People’s Hospital Affiliated to Nanchang University. This study was approved by the Ethics Committee (No: 20161009) of the People’s Hospital Affiliated to Nanchang University, Jiangxi Province, China. All procedures involving experimental animals were performed in accordance with protocols that were approved by the Committee for Animal Research of the People’s Hospital Affiliated to Nanchang University and complied with the *Guide for the Care and Use of Laboratory Animals* (NIH Publication No. 86‐23, revised 1985).

### IHC assay

A paraffin‐embedded tissue section was deparaffinized in xylene and rehydrated in graded series of EtOH followed by heat‐induced epitope retrieval in citrate buffer (pH 6.0). The section was incubated with anti‐Sox9 IgG (#82630, 1 : 200; Cell Signaling Technology, Danvers, MA, USA) at 4 °C overnight. A standard streptavidin‐biotin‐peroxidase complex method was used for staining, followed by counterstaining with Mayer’s hematoxylin. The IHC experiment was performed in triplicate using tumor tissue of similar size in each group.

### Statistical analysis

The analysis of the data was carried out using graphpad prism 7 (GraphPad Software, San Diego, CA, USA), and the data were presented as mean ± SD. One‐way ANOVA or two‐tailed Student’s *t*‐test was used to analyze the statistical difference. A *P* value <0.05 was regarded as having statistical significance.

## Results

### miR‐215‐5p is down‐regulated in breast carcinoma

**Figure 1:**
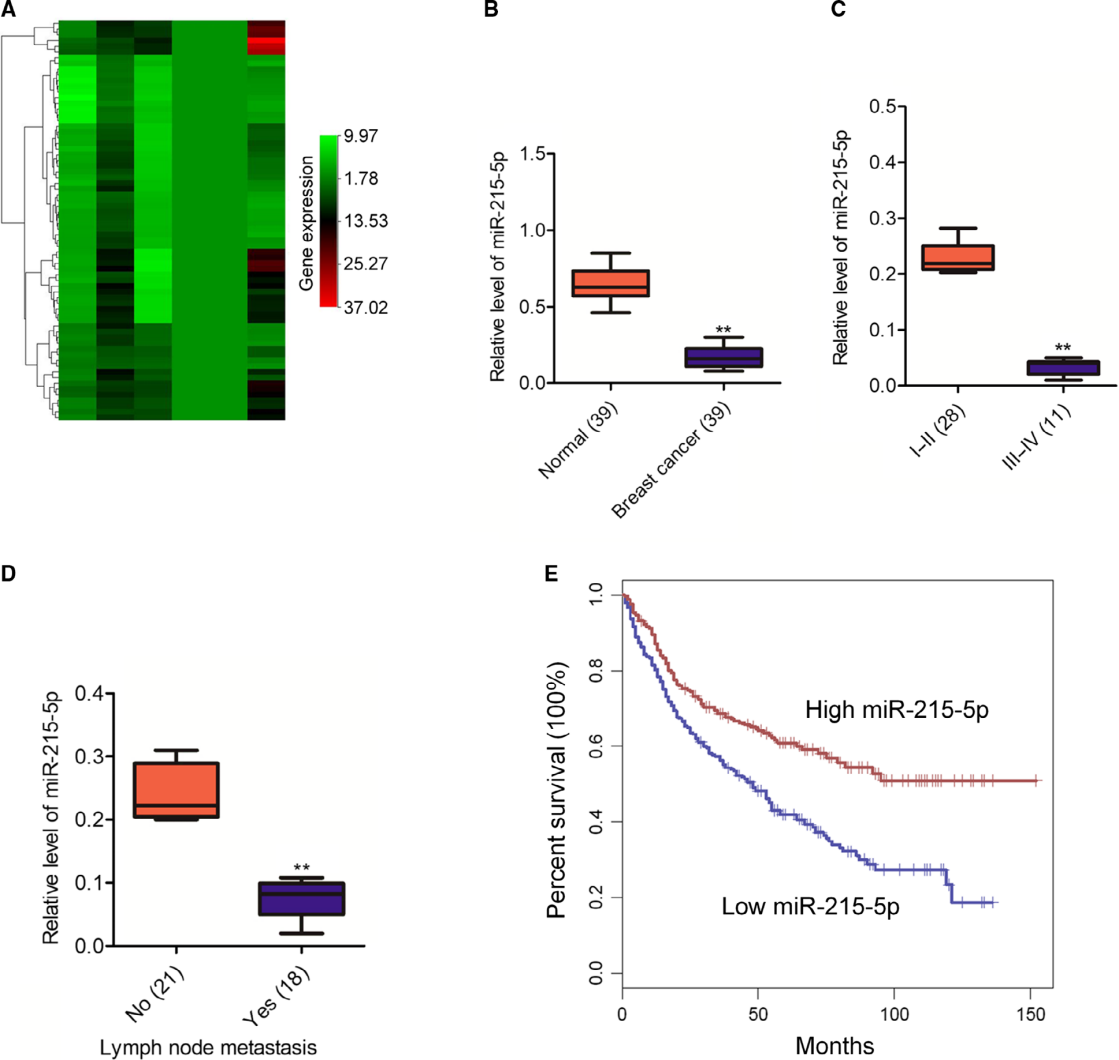
miR‐215‐5p is down‐expressed in breast carcinoma tissues. (A) Microarray analysis of miRNA expression in breast cancer tissues corresponding to normal tissues. (B) The expression of miR‐215‐5p in breast carcinoma and matched normal tissues was analyzed by using quantitative real‐time PCR. *n* = 39. **P < 0.01 compared with normal. (C) The levels of miR‐215‐5p in patients of different stages were detected via quantitative real‐time PCR. **P < 0.01 compared with stage I‐II. (D) The relationship of miR‐215‐5p and lymph node metastasis was analyzed by using quantitative real‐time PCR assay. **P < 0.01 compared with patients with no metastasis. All data are presented as mean ± SD (two‐tailed Student’s *t*‐test). (E) Kaplan‐Meier survival curves of patients with different levels of miR‐215‐5p.

First, we investigated the dysregulations of miRNAs in normal tissue and breast carcinoma tissue using Gene Expression Omnibus dataset GSE73002. The heatmap generated by the differential gene suggested that miR‐135‐5p was significantly down‐expressed in breast cancer tissues in comparison with the corresponding normal tissues (Fig. 1A). In addition, the analysis of the level of miR‐215‐5p was carried out in 39 pairs of breast cancer tissue and normal tissue by quantitative real‐time PCR assay. As shown in Fig. 1B, miR‐215‐5p was distinctly down‐expressed in breast carcinoma tissue. Furthermore, the level of miR‐215‐5p was allied to the advanced stage of breast cancer (Fig. 1C), as well as lymph node metastasis (Fig. 1D). Finally, the survival analysis implied that the lower level of miR‐215‐5p had an association with the unfavourable prognosis (Fig. 1E). These findings indicate that miR‐215‐5p is a suppressive miRNA in the progression of breast carcinoma.

### miR‐215‐5p restrains the aggressiveness of breast carcinoma

**Figure 2:**
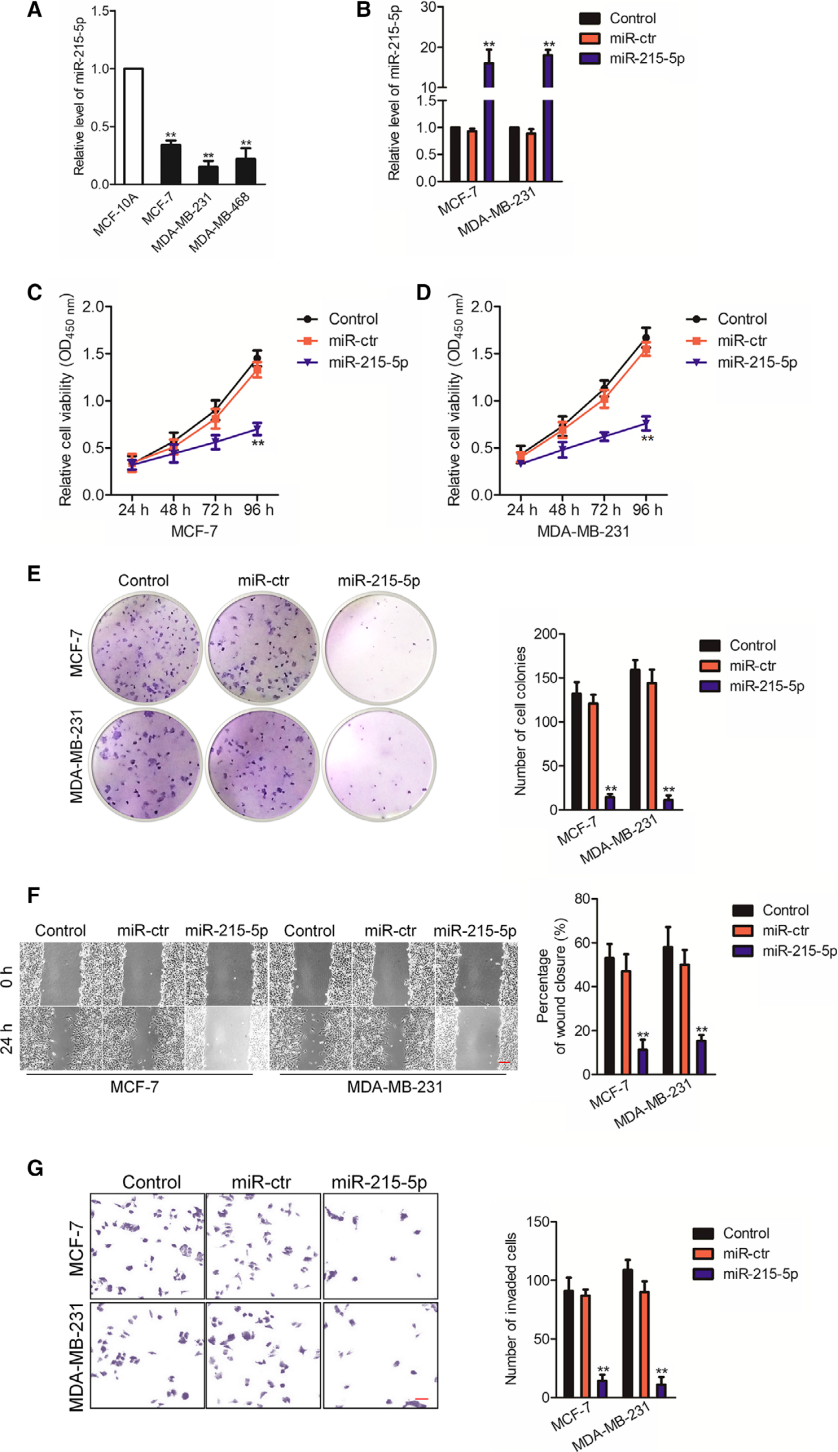
miR‐215‐5p suppresses growth, migration and invasion of breast cancer cells *in vitro*. (A) The expression of miR‐215‐5p in breast cancer cells and MCF‐10A cells was detected through quantitative real‐time PCR. (B) MDA‐MB‐231 and MCF‐7 cells were transfected with miR‐ctr or miR‐215‐5p mimic, and the level of miR‐215‐5p was detected by quantitative real‐time PCR. (C, D) The MTT assays were conducted to determine MDA‐MB‐231 and MCF‐7 cell viability. (E) Representative photographs of colon formation. (F) Cell migration was analyzed by using wound healing. Scale bars, 200 μm. (G) Invasion of cells was measured by using the Transwell invasion assay. *n* = 3. Scale bars, 200 μm. Data were expressed as mean ± SD (two‐tailed Student’s *t*‐test). **P < 0.01 compared with control.

Next, we analyzed the miR‐215‐5p levels in breast carcinoma cell lines and observed that miR‐215‐5p was down‐regulated in all breast carcinoma cells, in contrast with MCF‐10A (Fig. 2A). Next, miR‐215‐5p mimics were transfected into MCF‐7 and MDA‐MB‐231 cells to increase the endogenous level of miR‐215‐5p (Fig. 2B). miR‐215‐5p overexpression substantially suppressed the proliferation of MDA‐MB‐231 and MCF‐7 cells (Fig. 2C,D). Consistently, the colony formation of breast cancer cells that were transfected with miR‐215‐5p was dramatically reduced (Fig. 2E). In order to investigate whether miR‐215‐5p was involved in the metastatic‐related traits of breast cancer cells, we studied the function of miR‐215‐5p in breast cancer cell metastatic ability. The wound healing migration analysis verified that miR‐215‐5p substantially reduced the breast cancer cell migration (Fig. 2F). In addition, the invasive abilities of MCF‐7 and MDA‐MB‐231 cells were examined by Transwell invasion analysis, and the findings suggested that up‐regulation of miR‐215‐5p decreased the invasion of MCF‐7 and MDA‐MB‐231 cells (Fig. 2G). These findings imply that miR‐215‐5p represses the aggressiveness of breast carcinoma cells *in vitro*.

### Sox9 is the target of miR‐215‐5p

**Figure 3:**
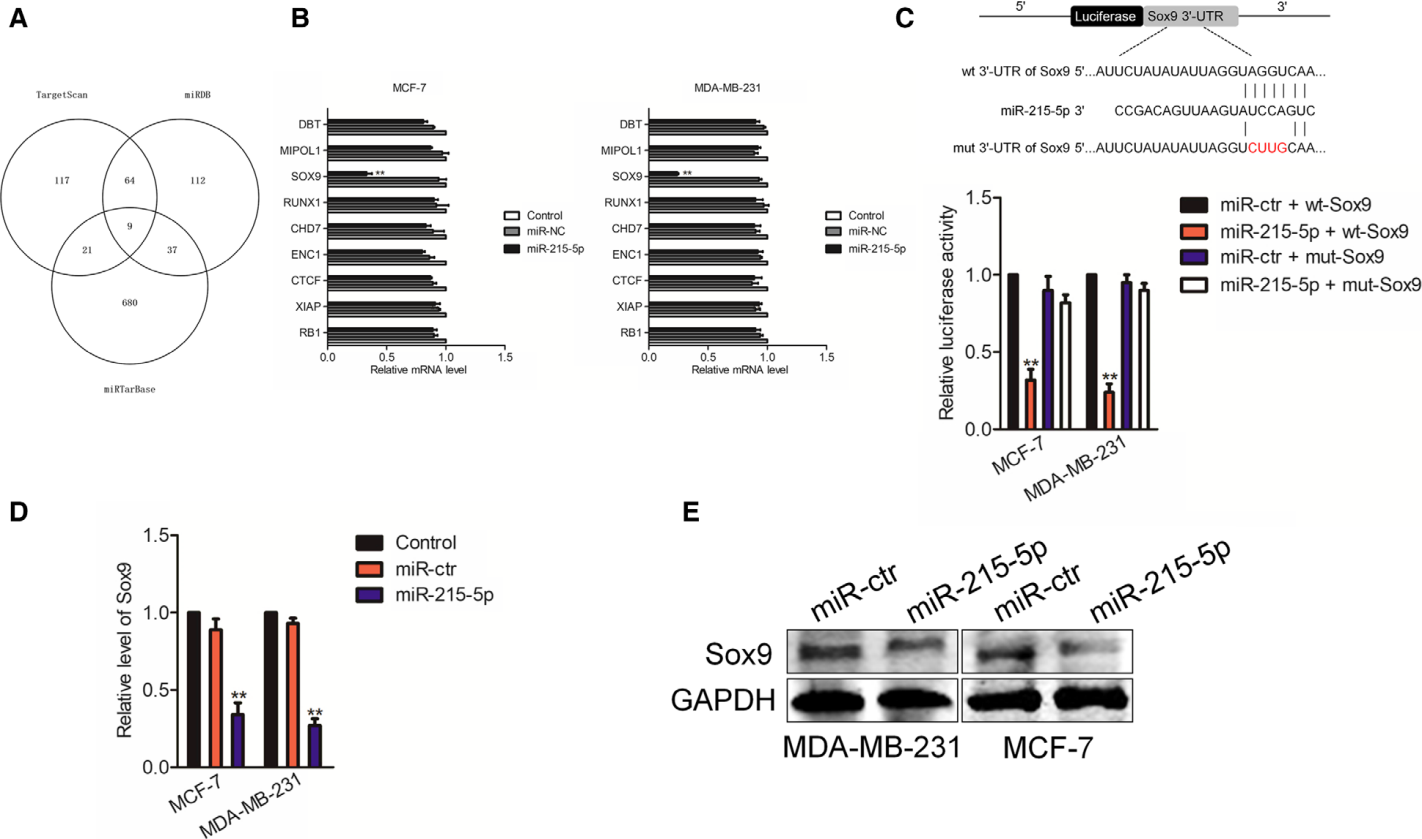
Sox9 is the target of miR‐215‐5p. (A) The complementary sequences of miR‐215‐5p were discovered in the 3'‐UTR of Sox9 mRNA using miRTarBase, miRDB and TargetScan. Venn graph represents the number of candidate common target genes determined by bioinformatics analysis. (B) MCF‐7 or MDA‐MB‐231 cells were transfected with miRNA negative control (miR‐NC) or miR‐215‐5p, and the levels of common target genes were detected using quantitative real‐time PCR. (C) The binding sites of miR‐215‐5p in the 3′‐UTR of Sox9 were predicted by using an online analysis tool, TargetScan. HEK‐293T cells were cotransfected with miR‐215‐5p and a pGL3 plasmid that contains wt or mutant 3′‐UTR of Sox9. The luciferase activities were detected. (D) The mRNA level of Sox9 in cells that transfected with miR‐215‐5p or miR‐ctr was measured. (E) The protein expression of Sox9 in miR‐ctr‐ or miR‐215‐5p‐transfected cells was detected by western blotting assay. *n* = 3. Data were expressed as the mean ± SD (two‐tailed Student’s *t*‐test). **P < 0.01 compared with control.

Next, the candidate targets of miR‐215‐5p were identified using miRDB, TargetScan and miRTarBase 13, 14, 15. As shown in Fig. 3A, a total of nine common target mRNAs was screened from three bioinformatics software tools. To find the target of miR‐215‐5p, we measured by quantitative real‐time PCR the levels of nine genes in MCF‐7 or MDA‐MB‐231 cells that were transfected with miR‐215‐5p. As shown in Fig. 3B, the level of Sox9 was reduced by miR‐215‐5p in both MDA‐MB‐231 and MCF‐7 cells. The binding site between miR‐215‐5p and the 3'‐UTR of Sox9 is shown in Fig. 3C. To explore the relationship between Sox9 and miR‐215‐5p, we carried out luciferase reporter gene analysis. We found that miR‐215‐5p reduced the luciferase activity in breast cancer cells transfected with the wt 3'‐UTR of Sox9 (Fig. 3C). Furthermore, miR‐215‐5p strikingly reduced the level of Sox9 as proved by both quantitative real‐time PCR and immunoblotting assay (Fig. 3D,E). These results imply that Sox9 is the target of miR‐215‐5p in breast cancer.

### The suppressive impact of miR‐215‐5p on breast carcinoma cells depends on Sox9

**Figure 4:**
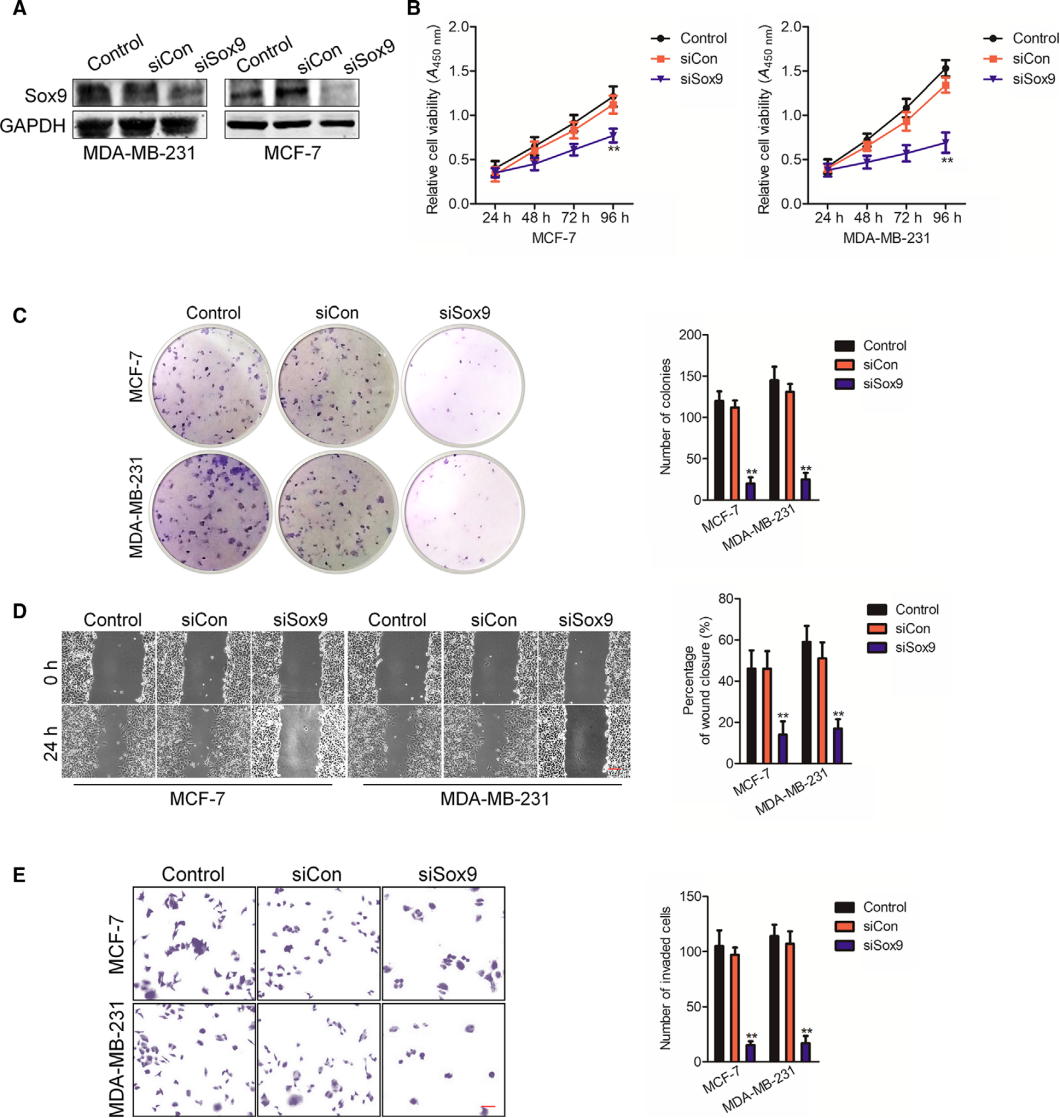
Sox9 knockdown phenocopies the suppressive effect of miR‐215‐5p. (A) MCF‐7 and MDA‐MB‐231 cells were transfected with siCon or siSox9. The expression of Sox9 was detected using western blotting assay. (B) The cell was transfected with siSox9 or siCon. MDA‐MB‐231 and MCF‐7 cell proliferation were determined through MTT assays. (C) Representative results of colony formation of MDA‐MB‐231 and MCF‐7 cells (left panel). The amount of colonies was counted. (D) Cells were transfected with siSox9 or siCon. The migration of MDA‐MB‐231 and MCF‐7 cells was detected using wound healing assay. Scale bars, 200 μm. (E) The invasion of cells was detected using Transwell invasion assay. *n* = 3. Scale bars, 200 μm. Data were expressed as the mean ± SD (two‐tailed Student’s *t*‐test). **P < 0.01 compared with control.

Previous findings illustrated that miR‐215‐5p negatively regulates the expression of Sox9 through binding with the 3'‐UTR of Sox9. Accordingly, as we speculated, miR‐215‐5p mediated the progression of breast cancer cells via regulating Sox9. siSox9 was applied for reducing the expression of Sox9 (Fig. 4A), and we explored the down‐regulation of Sox9 on the development of breast cancer cells. As shown in Fig. 4B, Sox9 silencing suppressed the proliferation of MDA‐MB‐231 and MCF‐7 cells. Meanwhile, the colony formation assay proved that down‐regulation of Sox9 substantially lowered the colony formation in breast cancer cells (Fig. 4C). Furthermore, we investigated the effect of knocked‐down Sox9 on the aggressiveness of breast carcinoma cells. As shown in Fig. 4D,E, Sox9 silencing significantly impaired the migration and invasive phenotype of MDA‐MB‐231 and MCF‐7 cells.

### The inhibitory impact of miR‐215‐5p is rescued by Sox9

**Figure 5:**
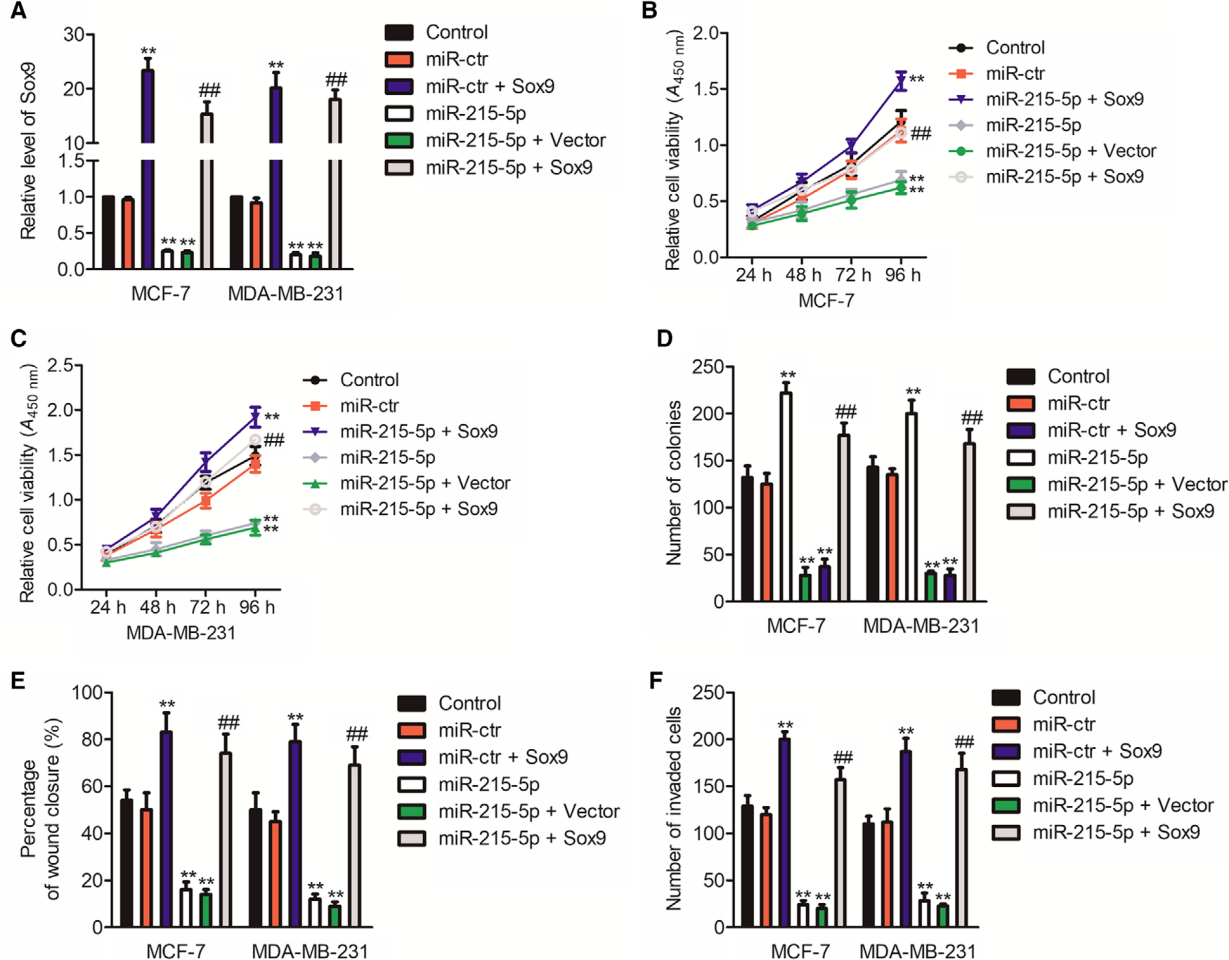
Sox9 is a functional target of miR‐215‐5p. (A) Breast cancer cells were transfected with miR‐215‐5p alone or cotransfected with miR‐215‐5p + Sox9‐expressing plasmid. The level of Sox9 was detected by quantitative real‐time PCR assay. (B, C) A breast cancer cell was transfected with miR‐215‐5p alone or cotransfected with miR‐215‐5p + Sox9‐expressing plasmid. Cell viability was detected through MTT assay. (D) Representative result of colony formation in MDA‐MB‐231 and MCF‐7 cells. (E) Cell was transfected with miR‐215‐5p alone or cotransfected with miR‐215‐5p + Sox9‐expressing plasmid. The migration of cells was detected using wound healing assay. (F) The invasion of cells was determined by Transwell invasion assay. *n* = 3. Data were expressed as the mean ± SD (two‐tailed Student’s *t*‐test). **P < 0.01 compared with control; ^##^ *P* < 0.01 compared with the miR‐215‐5p group.

Next, breast cancer cells were transfected in combination with miR‐215‐5p and Sox9 to demonstrate that Sox9 was the functional target of miR‐215‐5p (Fig. 5A). Re‐expression of Sox9 significantly attenuated the inhibition impact of miR‐215‐5p on the proliferation and colony formation of breast cancer cells (Fig. 5B–D). Furthermore, Sox9 overexpression partially offsets the inhibition impact of miR‐215‐5p on the invasion and migration of MCF‐7 and MDA‐MB‐231 cells (Fig. 5E,F). As revealed by all of these data, the inhibitory impact of miR‐215‐5p on the malignant phenotypes of breast cancer cells is rescued by Sox9.

### miR‐215‐5p suppresses the progression of breast carcinoma cells *in vivo*

**Figure 6:**
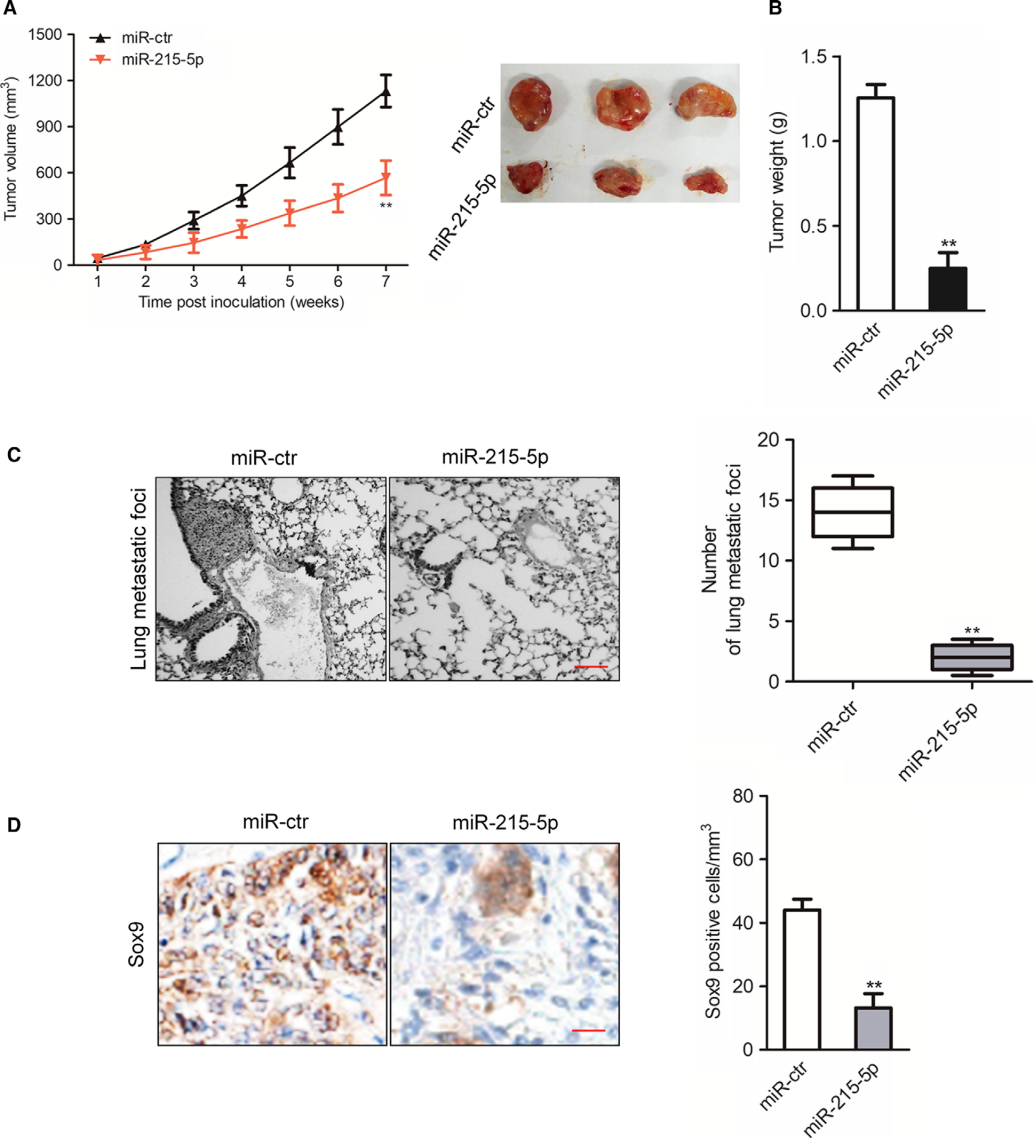
Overexpression of miR‐215‐5p suppresses MDA‐MB‐231 cell growth and metastasis. (A, B) Mice were injected subcutaneously with miR‐ctr‐ or miR‐215‐5p‐transfected MDA‐MB‐231 cells. The tumor volumes and tumor weights of the subcutaneous breast cancer cell implantation model. (C) Nude mice were injected with miR‐215‐5p‐ or miR‐ctr‐transfected MDA‐MB‐231 cells through tail veins. The number of metastatic foci on the lung from mice injected with MDA‐MB‐231 cells was counted. Scale bars, 200 mm. (D) Representative results of immunohistochemical analyses of Sox9 in tumor tissues. *n* = 3. Scale bars, 200 μm. Data were expressed as the mean ± SD (two‐tailed Student’s *t*‐test). **P < 0.01 compared with miR‐ctr.

Finally, the xenograft tumor model was constructed by subcutaneous inoculation of miR‐215‐5p‐ or miR‐ctr‐transfected MDA‐MB‐231 cells into nude mice. We observed that tumors formed by miR‐215‐5p‐overexpressing MDA‐MB‐231 cells grew slower as compared with the control group (Fig. 6A,B). Then, the lung metastasis model was constructed by injecting MDA‐MB‐231 cells into nude mice. In those mice, which were injected with miR‐215‐5p‐overexpressing MDA‐MB‐231 cells, the lung metastasis rate of MDA‐MB‐231 cells was significantly reduced in comparison with the control group (Fig. 6C). Similar to the impact of miR‐215‐5p on Sox9 expression *in vitro*, miR‐215‐5p decreased the expression of Sox9 *in vivo* (Fig. 6D). In conclusion, miR‐215‐5p inhibits the development of breast cancer cells *in vivo*.

## Discussion

In spite of the advanced progress made in treating breast cancer, the survival rate of patients remains unsatisfactory 16, 17. Therefore, new prognostic markers and treatment options are urgent for the improvement of medical treatment for patients with breast cancer. Previous investigations have determined that miR‐215 serves as an anti‐oncogene in several kinds of cancers, but the mechanisms by which miR‐215‐5p is involved in breast carcinoma progression have not been effectively studied 9, 11, 18, 19. This current study reveals that miR‐215‐5p is down‐regulated in breast cancer and is negatively correlated with the progression and metastasis of breast carcinoma. Up‐regulation of miR‐215‐5p inhibits the growth, migration and invasion of breast cancer cells. In addition, Sox9 is identified as the functional target of miR‐215‐5p. Knockout of Sox9 mimicked the inhibitory impact of miR‐215‐5p on breast carcinoma cells. As revealed by these results, miR‐215‐5p serves as a cancer suppressor in human breast cancer.

The investigation of the fundamental function of miR‐215 in the progression of malignant tumors has been carried out in preclinical research 20. Herein, we discovered that miR‐215‐5p was significantly downregulated in breast cancer compared with noncancerous tissue. In line with the earlier research, we discovered that the expression of miR‐215‐5p was inversely correlated with the progression and metastasis of breast cancer. Importantly, the prognostic analysis proved that the clinical outcomes of patients who had a high level of miR‐215‐5p were superior to those of patients who had a lower miR‐215‐5p level.

The aberrant expression of miR‐215‐5p performs the regulation of multiple cancer‐related signaling pathways, for instance, cell growth, invasion and metastasis. As revealed by our findings, miR‐215‐5p substantially suppressed not just the growth but also the migration and invasion of breast cancer cells. These findings were consistent with the previous report, in which miR‐215‐5p inhibits the progression of colon cancer by modulating epidermal growth factor receptor ligand and Homeobox protein Hox‐B9 20. In contrast, miR‐215‐5p had the potential of inhibiting the mRNA and the protein expression of Protocadherin‐9 in glioma cells through regulating its promoter and 3'‐UTR 21. The luciferase reporter gene assay and western blotting assay demonstrated that Sox9 is the potential target of miR‐215‐5p in breast cancer. Meanwhile, overexpression of Sox9 converted the suppressive impact of miR‐215‐5p on the growth and metastatic‐related traits of breast cancer cells, suggesting that Sox9 is the downstream target of miR‐215‐5p. In this investigation, we also demonstrated that miR‐215‐5p overexpression decreased tumor development and distant metastasis of breast carcinoma cells in nude mice.

Substantive reports have confirmed that the epithelial‐mesenchymal transition process contributes to the metastasis of several cancers, including breast cancer 22, 23, 24. Even though our tests suggest that miR‐215‐5p overexpression blocks the aggressive phenotypes of cancer cells, whether miR‐215‐5p mediates breast cancer metastasis and is associated with regulating the epithelial‐mesenchymal transition process continues to be unknown. Therefore, more in‐depth studies are required to reveal other downstream signaling pathways, in addition to key regulatory processes of miR‐215‐5p in the progression of breast cancer.

In conclusion, our study proves that miR‐215‐5p is down‐regulated in human breast carcinoma, and the level of miR‐215‐5p has an inverse relationship with the metastasis and prognosis of patients with breast cancer. In addition, miR‐215‐5p is a suppressive miRNA in the progression of breast cancer by regulation of Sox9 expression.

## Conflict of interest

The authors declare no conflict of interest.

## Author contributions

JBG and MNZ conducted experiments and were responsible for data acquisition, analysis, interpretation and manuscript writing. XLZ conceived and designed the study. All authors read and approved the final manuscript.

## Supporting information


**Table S1.** Characteristics of patients with breast cancer in the present study.
