# Supplementary material for: miRNA‐215‐5p suppresses the aggressiveness of breast cancer cells by targeting Sox9
Source: FEBS Open Bio. 2019 Oct 22;9(11):1957–67. doi: 10.1002/2211-5463.12733 (PMC6823282; doi:10.1002/2211-5463.12733)
Supplement: Supplementary file 1 — Table S1. Characteristics of patients with breast cancer in the present study. [file FEB4-9-1957-s001.docx]

**Supplementary Table 1.** Characteristics of patients with breast cancer in the present study.

| **Variables** | **MiR-215-5p** | | ***P*-value** |
| --- | --- | --- | --- |
|  | High (n) | Low (n) |  |
| **Age (years)** |  |  | 0.13 |
| <50 | 8 | 10 |  |
| >50 | 13 | 9 |  |
| **Tumor size** |  |  | 0.27 |
| ≧2cm | 11 | 8 |  |
| <2cm | 7 | 13 |  |
| **LNM** |  |  | <0.01 |
| Yes | 5 | 13 |  |
| No | 17 | 4 |  |
| **Histology grade** |  |  | 0.33 |
| Well | 5 | 6 |  |
| Moderate | 8 | 12 |  |
| Poor | 3 | 5 |  |
| **TNM stage** |  |  | <0.01 |
| I-II | 20 | 8 |  |
| III-IV | 3 | 8 |  |
| **ER status** |  |  | 0.41 |
| Negative | 10 | 6 |  |
| Positive | 13 | 10 |  |
| **PR status** |  |  | 0.47 |
| Negative | 11 | 15 |  |
| Positive | 6 | 7 |  |
| **HER2 status** |  |  | 0.34 |
| Negative | 12 | 8 |  |
| Positive | 9 | 10 |  |

ER, estrogen receptor; HER2, human epidermal growth factor receptor-2; LN, lymph node; PR, progesterone receptor.
